# Pembrolizumab-Induced Secondary Cholangitis: A Case Report

**DOI:** 10.7759/cureus.98586

**Published:** 2025-12-06

**Authors:** Nur Aisyah Muhd Opandi, Aqeel Ahmed, Emma Robinson, Answar Abdulrehman

**Affiliations:** 1 Internal Medicine, Chesterfield Royal Hospital NHS Foundation Trust, Chesterfield, GBR; 2 Gastroenterology, Chesterfield Royal Hospital NHS Foundation Trust, Chesterfield, GBR

**Keywords:** acute cholangitis, immune check-point inhibitor, immunotherapy, immunotherapy-related adverse events, non-small cell lung carcinoma (nsclc), pembrolizumab, pembrolizumab side effect, primary sclerosing cholangitis (psc)

## Abstract

Immune checkpoint inhibitors (ICIs) are increasingly being used as part of cancer treatment. Whilst effective oncological therapies, these drugs are associated with a significant risk of immune-related adverse events (IrAEs). They can affect a variety of organ systems, including the lungs, heart, kidneys, and gastrointestinal tract, with liver damage being a relatively well-recognised complication. There have been multiple previous reports describing immune-related hepatitis; however, more recent studies are starting to describe inflammation within the extrahepatic and intrahepatic bile ducts, specifically discussing immune-related cholangitis. This can be in addition to or distinct from hepatitis. Immune-related cholangitis remains rare and less extensively studied than other complications; however, it represents a significant adverse event that may evolve into potentially severe complications such as liver failure and can be fatal. We present a case of a 50-year-old man with non-small cell lung carcinoma (NSCLC) with brain metastases, who developed immune-related cholangitis following 11 cycles of the immune checkpoint inhibitor pembrolizumab. The patient presented with severe abdominal pain and transaminitis. A comprehensive workup excluded bacterial, viral, autoimmune and other alternative aetiologies leading up to the diagnosis of ICI-induced cholangitis, based on magnetic resonance cholangiopancreatography (MRCP). The patient responded well to the combination of ursodeoxycholic acid (UDCA), corticosteroid therapy and mycophenolate mofetil (MMF), showing gradual improvement in liver enzyme levels and significant improvement in abdominal pain. At the time of writing this case report, subsequent slow steroid weaning has been successful, without clinical or biochemical flare. As the use of ICIs increases, this case highlights the importance of awareness of immune-related cholangitis as a rare but significant adverse event to enable a timely diagnosis and treatment. Immune-related cholangitis such as this is crucial to identify as distinct from infective cholangitis, as treatment is primarily immunosuppression. It adds to the growing body of evidence of immune-related cholangitis as a specific adverse reaction, encouraging and enabling more research into this to guide future clinical decision-making and the formation of treatment guidance and diagnostic criteria.

## Introduction

Immune checkpoint inhibitors (ICIs) have changed cancer treatment options dramatically in recent years [1]. The immune-checkpoint protein programmed cell death-1 (PD-1) was identified in 1992, and subsequent development of immunotherapies targeting this protein has allowed for major advancements in cancer treatment [1]. Pembrolizumab is a human IgG4 monoclonal antibody against PD-1, blocking this immune checkpoint and inhibiting part of the immunosuppressive repertoire of cancer cells. Pembrolizumab is currently used for a variety of different cancers, including non-small cell lung carcinoma (NSCLC), melanoma, head and neck squamous cell carcinoma, breast cancer, gynaecological, renal, haematological, and gastrointestinal malignancies. Respiratory cancers appear to be the most common treatment indication when looking at reported patients with immune-related cholangitis, making up around 40% of reported cases [2].

Whilst improving survival benefits for patients, these medications are associated with significant adverse reactions, which are becoming increasingly common as use of ICIs increases [3]. Immune-related adverse events (IrAEs) have been demonstrated to affect multiple organ systems, including the respiratory, cardiovascular, gastrointestinal and nervous systems [2]. The liver has been a well-documented affected site, with pembrolizumab-induced hepatitis being a well-recognised complication, occurring in up to 16% of patients; however, cholangitis is less common and less well studied, reported in only 0.05%-0.73% of patients, with limited existing literature exploring this [4, 5]. Immune-related cholangitis is a form of cholangitis thought to be similar to primary sclerosing cholangitis (PSC), characterised by pain and inflammation through CD8-positive T cell infiltration into the bile ducts [6]. Abdominal pain, a rise in liver enzymes and non-obstructive biliary dilatation appear to be common amongst cases [7], matching the clinical picture in our patient. This can be hard to diagnose, as the clinical picture is difficult to distinguish from autoimmune or infective cholangitis. 

This case report presents a case of a patient with metastatic NSCLC who developed immune-related cholangitis following 11 cycles of treatment with pembrolizumab. There was a delay in this diagnosis, in part due to non-specialist clinicians not having an awareness of the side effects of these specialist medications. This case emphasises the importance of awareness and recognition of this rare adverse event to facilitate early diagnosis and treatment, as delays in recognition or treatment may potentially result in progression to liver failure. Our aim is to highlight the need for close monitoring and guide future research to adapt diagnostic and treatment guidelines of irAE-induced cholangitis in patients given ICI therapy.

## Case presentation

We present the case of a 50-year-old male patient who presented to a district general hospital with a two-day history of severe epigastric and right upper quadrant abdominal pain associated with severe nausea and bilious vomiting. He reported a preceding six-week history of intermittent abdominal pain. He had a background of stage 4 metastatic NSCLC with previous craniotomy and resection of brain metastases and small-volume hilar and mediastinal lymphadenopathy. His only other comorbidity was hypertension. He had no clinically significant genetic mutations, and PDL1 testing had shown the tumour proportion score above 50%. Palliative pembrolizumab was started as single-agent therapy, and he received 11 cycles in total between November 2023 and January 2025. His last course had ended 11 weeks prior to this presentation. On initial clinical examination, the patient was stable with no fever, no clinical evidence of jaundice, but with marked epigastric tenderness. His regular medication was reviewed, and none were noted to be hepatotoxic. 

At initial presentation, laboratory investigations (Table 1) showed significantly increased alkaline phosphatase (ALP) levels at 537 U/L, with raised aspartate transaminase (AST) at 71 U/L and alanine transaminase (ALT) at 281 U/L, and slightly raised C-reactive protein (CRP) at 29 mg/L. Bilirubin was normal at 8 μmol/L.

**Table 1:** Blood tests on admission ALP: alkaline phosphatase; ALT: alanine aminotransferase; AST: aspartate aminotransferase; GGT: gamma-glutamyl transferase; WCC: white cell count; CRP: C-reactive protein; U/L: units per litre; μmol/L: micromoles per litre; mg/L: milligrams per litre; mmol/L: millimoles per litre

Parameter	Result	Referance Range
ALP	537 U/L	30 - 130 U/L
ALT	281 U/L	7 - 55 U/L
AST	71 U/L	0 – 40 U/L
Bilirubin	8 μmol/L	5 - 21 μmol/L
GGT	533 U/L	5 - 50 U/L
AST:ALT ratio	0.7 U/L	-
Amylase	115 U/L	30-118 U/L
WCC	8.62 10^9/L	4.0 - 11.0 x 10*9/L
CRP	29 mg/L	<5 mg/L
Lactate	0.8 mmol/L	0.5 - 2.2 mmol/L

A comprehensive autoimmune panel, including serum immunoglobulin (Ig) as well as Ig subclasses of IgG1, IgG2, IgG3, and IgG4, and liver autoantibodies (mitochondrial antibody, smooth muscle antibody, and liver kidney microsomal antibody), was unremarkable, thus ruling out autoimmune hepatitis as a diagnosis. Antinuclear antibody (ANA) was negative. The patient was found to have p-ANCA weak positivity; however, normal myeloperoxidase (MPO) antibody ANCA and normal proteinase 3 (PR3) antibody ANCA, making PSC unlikely (Table 2). An extensive infectious panel (Table 3), including cytomegalovirus (CMV), Epstein-Barr virus (EBV), human immunodeficiency virus (HIV), hepatitis B, and hepatitis C, was negative; therefore, we were able to exclude major hepatotropic and herpes viruses as causes.

**Table 2:** Autoimmune and antibody panel IgA: immunoglobulin A; IgG: immunoglobulin G; IgM: immunoglobulin M; ANA: antinuclear antibody; P-ANCA: perinuclear anti-neutrophil cytoplasmic antibodies; ANCA: anti-neutrophil cytoplasmic antibodies; g/L: grams per litre; IU/mL: international units per millilitre

Parameter	Result	Reference Range
IgA	3.69 g/L	0.8 - 4 g/L
IgG	8 g/L	6 - 16 g/L
IgM	0.54 g/L	0.5 - 2 g/L
IgG1	5.15 g/L	3.2 - 10.2 g/L
IgG2	0.9 g/L	1.2 - 6.6 g/L
IgG3	0.21 g/L	0.2 - 1.9 g/L
IgG4	0.06 g/L	0 - 1.3 g/L
Mitochondrial antibody	Negative	-
Smooth muscle antibody	Negative	-
Liver-kidney microsomal antibody	Negative	-
ANA	Negative	-
p-ANCA	Weak positive	-
Myeloperoxidase (MPO) antibody ANCA	0.7 IU/mL	<3.5 IU/mL
Proteinase 3 (PR3) antibody ANCA	1.5 IU/mL	<2 IU/mL

**Table 3:** Infectious panel report CMV: cytomegalovirus; EBV: Epstein–Barr virus; HIV: human immunodeficiency virus

Parameter	Result
CMV DNA	Not detected
EBV DNA	Not detected
HIV	Not detected
Hepatitis B surface antigen	Not detected
Hepatitis C antibody	Not detected

**Figure 1:**
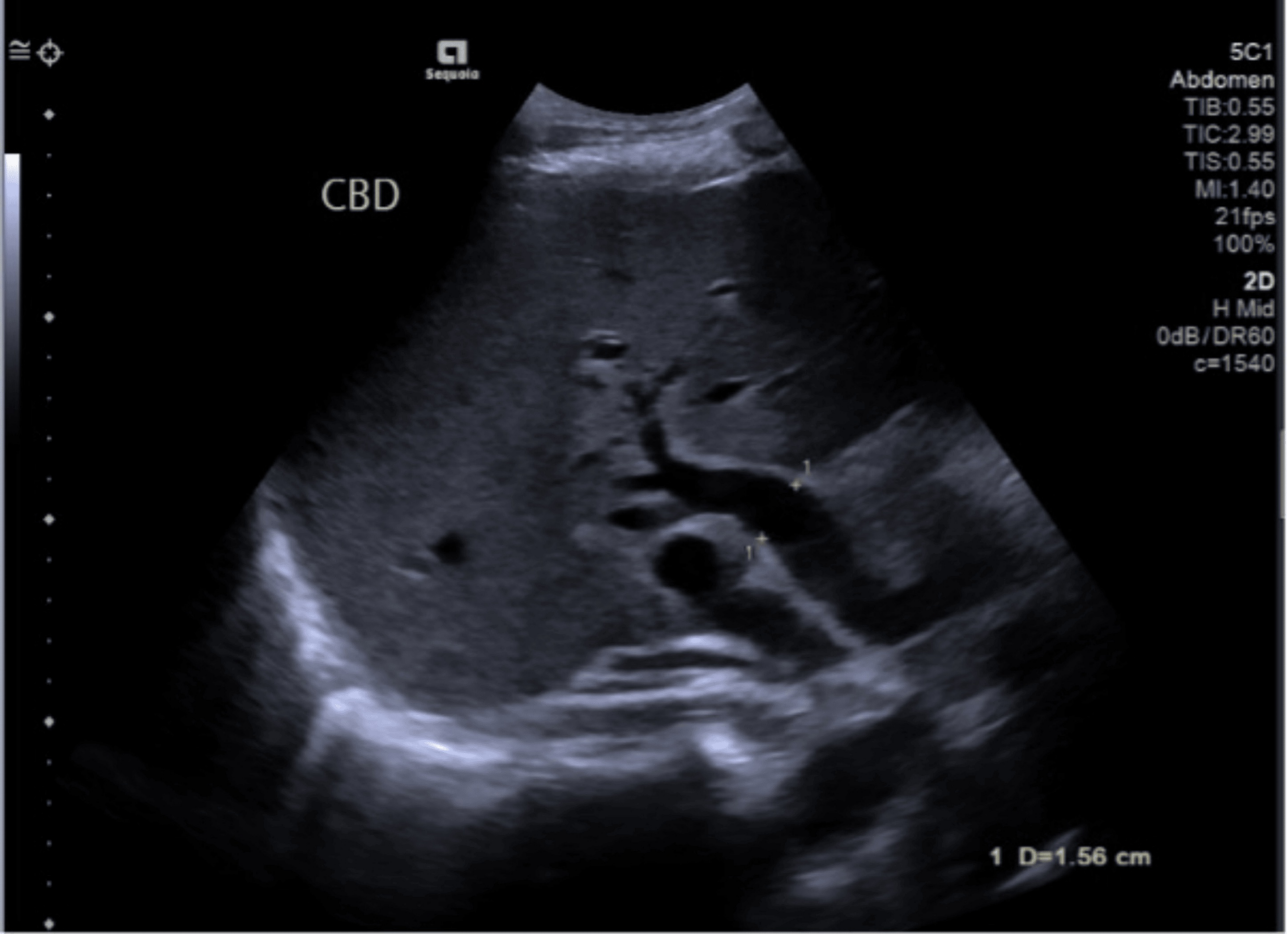
An ultrasound of the abdomen showing measured CBD dilatation with no calculi CBD: common bile duct

An urgent computed tomography (CT) scan of the chest, abdomen and pelvis with contrast was done, and this ruled out a liver disease progression but revealed a thick-walled gallbladder and common bile duct (CBD) dilation to 15 mm, with intrahepatic biliary dilatation and enhancement of the CBD wall. These appearances were suspicious of acute cholangitis and cholecystitis. An ultrasound of the abdomen (Figure 1) was performed the following day, which confirmed the CT results. The gallbladder had a thickened, oedematous wall consistent with acute cholecystitis, with dilatation of the CBD and minor intrahepatic biliary duct dilatation; however, it was noted that there were no biliary or CBD calculi, which made the diagnosis of cholecystitis less likely.

**Figure 2:**
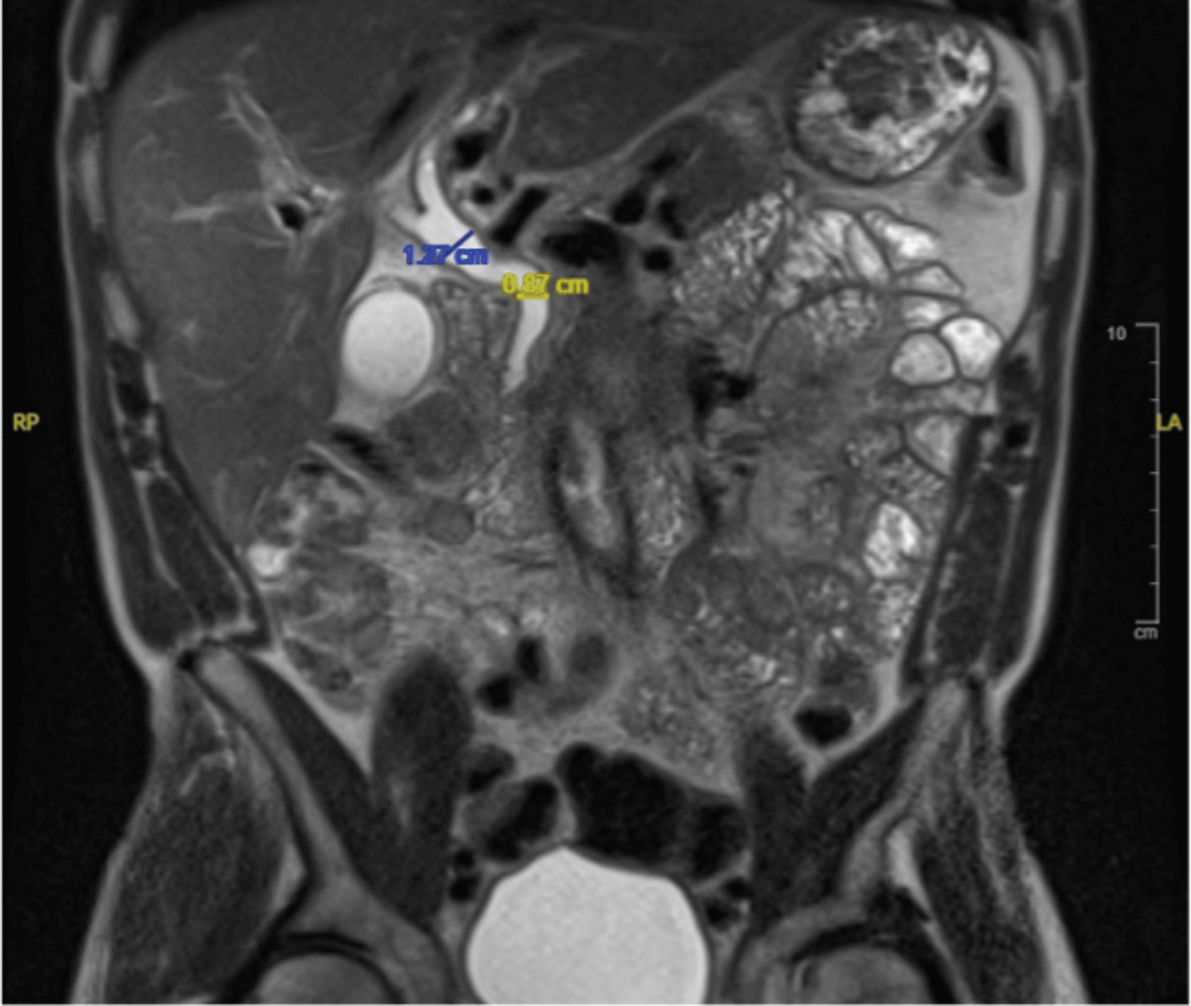
MRCP findings demonstrate common hepatic duct distension (blue) and thickening of the distal common bile duct wall with pericholecystic fluid consistent with acute cholangitis. MRCP: magnetic resonance cholangiopancreatography

Magnetic resonance cholangiopancreatography (MRCP) was then arranged, which demonstrated mildly dilated intra- and extrahepatic ducts and foci of distention in the common hepatic duct, with some thickening of the distal CBD wall and evidence of pericholecystic fluid (Figure 2). These findings were consistent with acute cholangitis (in view of the biliary enhancement on recent CT, smooth thickening of the wall, and global distension), with associated acalculous cholecystitis. 

Oesophagogastroduodenoscopy (OGD) was performed to exclude any other causes for the patient's symptoms, such as any periampullary lesion. This only showed gastritis in the gastric body and a 3cm hiatus hernia, which were not thought to be causing the symptoms described. Biopsy of the gastric body found mild changes of reactive gastropathy. 

Given the timing of symptom onset, exclusion of alternative causes, and MRCP findings, the most likely clinical diagnosis was thought to be acalculous cholangitis secondary to recent immunotherapy.

**Figure 3:**
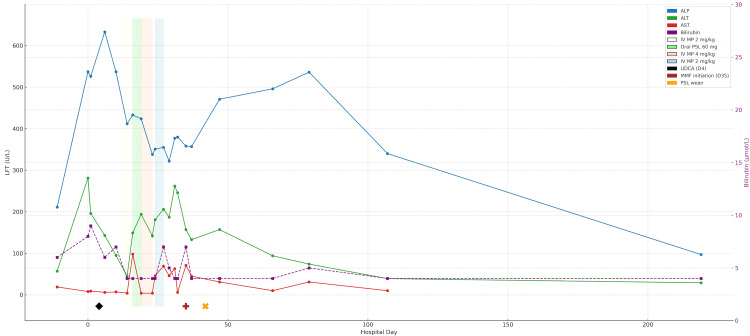
Longitudinal levels of serum ALT, AST, ALP and bilirubin during hospitalization in relation to treatment LFT: liver function test; ALP: alkaline phosphatase; AST: aspartate aminotransferase; ALT: alanine aminotransferase; IV: intravenous; MP: methylprednisolone; PSL: prednisolone; UDCA: ursodeoxycholic acid; MMF: mycophenolate mofetil; U/L: units per litre; μmol/L: micromoles per litre; mg/kg: milligram per kilogram.

The patient was initially treated with intravenous co-amoxiclav and gentamicin for suspected infective cholangitis, which did not improve his clinical picture, and his liver enzymes continued to rise. This was later deemed unlikely due to the absence of fever or a significant rise in CRP (Table 4). He was started on ursodeoxycholic acid (UDCA) at 12 mg/kg/day on Day 5 of admission, and this showed initial brief improvement to his liver enzymes (Figure 3). Based on MRCP findings and high suspicion of immune-mediated cholangitis, a multidisciplinary discussion involving the local specialist team, including the radiology, oncology and hepatology teams, was conducted. Hence, at Day 14 of admission, an empirical methylprednisolone was commenced at 2 mg/kg/day. Daily monitoring of liver function tests (LFT) initially revealed a stable picture of ALT, ALP and AST with IV methylprednisolone. Due to this, he was subsequently converted to oral prednisolone 60 mg once daily. However, after the 4^th^ day of oral steroid treatment, a worsening trend of liver function (ALT 194 U/L, ALP 424 U/L) was seen, and steroids were again increased to IV methylprednisolone given at 4 mg/kg/day. The transaminitis trend showed to be progressive and fluctuant with corticosteroids, but at worst was up to grade 2 transaminitis (Figure 3). Considering corticosteroid refractoriness, he was started on mycophenolate mofetil (MMF) 500mg twice a day, and this eventually showed a good response and gradual improvement in his liver enzymes. Subsequent slow steroid weaning has been successful, without clinical or biochemical flare at the time of writing. A repeat abdominal ultrasound was performed and showed a reduction in the degree of dilatation and wall thickness in comparison to previous imaging.

**Table 4:** Trend of CRP during hospitalisation CRP: C-reactive protein; mg/L: milligrams per litre

Day of hospitalisation	CRP level (mg/L)
Day 0	29
Day 1	36
Day 3	28
Day 4	26
Day 5	36
Day 9	34
Day 13	35
Day 14	21
Day 16	38
Day 17	36

Clinically, the patient's abdominal pain significantly improved following treatment, and the patient remained asymptomatic. Due to clinical complexity, multidisciplinary discussion with specialists from different areas, including radiologists, oncologists and hepatologists, was involved early to support management of these serious irAEs, and the patient was subsequently transferred from our district general hospital to a tertiary hepatology centre. 

## Discussion

Pembrolizumab-based therapy is a valuable first-line treatment option for advanced or metastatic NSCLC. Pembrolizumab strongly inhibits the PD-1/PD-L1 immune signalling pathway and thus is known to be one of the most successful new therapies for cancer [8]. Despite this important breakthrough, an objective response to immunotherapy only reaches approximately 20% of patients with advanced NSCLC, and pembrolizumab was associated with specific adverse events [9].

In a recent study, looking specifically at documented cases of ICI-associated cholangitis, Tan et al. emphasise that this is an underreported phenomenon and highlight the importance of identifying specific cases of cholangitis, as distinct from ICI-associated hepatitis, as it is often more broadly classified as ICI-related liver injury [2]. They suggested cholangitis has been shown to be clinically distinct, with a more prolonged latent period when compared to ICI-induced hepatitis. Studies have shown delayed onset of symptoms up to two years after treatment with a median time to onset of 77 days compared to 44 days for ICI-induced hepatitis, although the mechanisms of this are not well understood, and further research is required [2].

**Figure 4:**
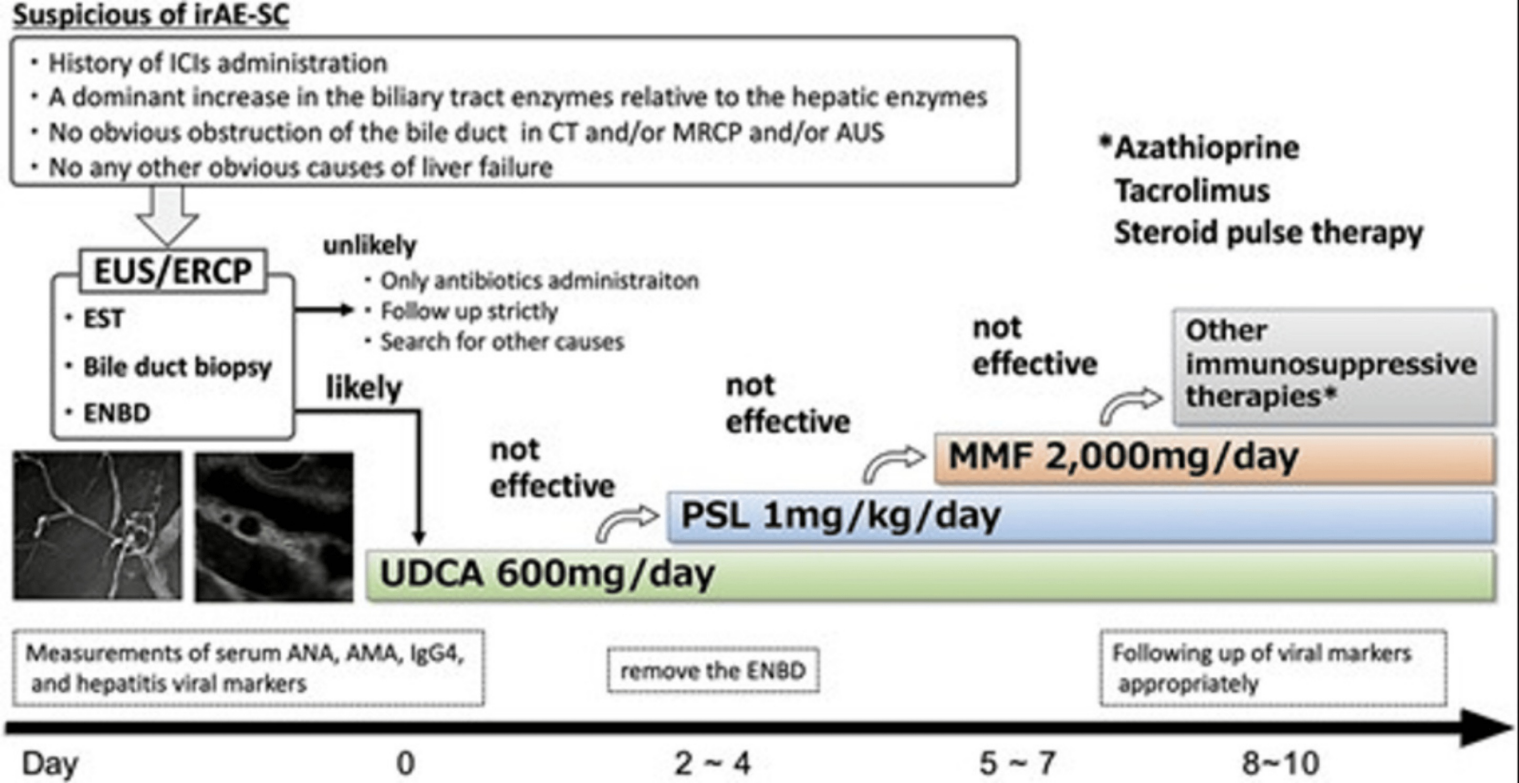
Our treatment strategy for irAE-SC. ICI: immune checkpoint inhibitor; CT: computed tomography; AUS: abdominal ultrasonography; EUS: endoscopic ultrasonography; EST: endoscopic sphincterotomy; ENBD: endoscopic nasobiliary drainage; UDCA: ursodeoxycholic acid; PSL: prednisolone; MMF: mycophenolate mofetil; ANA: antinuclear antibody; AMA: antimitochondrial antibody; IgG4: immunoglobulin G4; irAE: immune-related adverse event; SC: secondary cholangitis

Immune-related cholangitis is a rare type of hepatic irAE that has only been reported in 0.05-0.73% of patients, and much remains unknown about its optimal treatment [6]. Okamoto et al. [6] outline a treatment protocol (Figure 4) starting with UDCA prior to steroid treatment and escalating to further immunosuppressive treatments if required. 

This needs to be expanded further, with larger clinical studies, in order to create formal treatment guidance and diagnostic criteria. Most studies show limited response to immunosuppressive agents, including steroid therapy, and sadly, the prognosis of these patients remains poor [6,7]. The European Society for Medical Oncology currently recommends the use of UDCA alongside steroids for this condition, but evidence is limited, and evidence currently suggests that most cases of ICI-induced cholangitis have a poor response to steroids [2]. 

This patient presented with abdominal pain and elevated liver enzymes, which could be seen in various hepatic pathologies, including viral hepatitis, autoimmune hepatitis, drug-induced liver injury, or progression of disease. There was a delay in the diagnosis due to clinical complexity; however, given the timing of the patient’s symptoms with prolonged latency of 77 days post treatment, similar to recent studies [2], the exclusion of other differential diagnoses through comprehensive workup and multidisciplinary discussion, thus a diagnosis of ICI-induced cholangitis was made. In our case, the patient showed fluctuant and non-persistent improvement with UDCA and corticosteroids, which fits with a previous study that suggests poor steroid response. He only demonstrated complete improvement in the cholestatic injury after being treated with a combination of MMF.

This case adds to the growing body of reports of this very rare complication, in the hope that clinicians will be able to recognise this clinical picture in the future and that it will contribute to the broader scientific literature. Case reports like this will help to guide future research, enabling adaptation of specific treatment guidelines and diagnostic criteria. Further research also helps to provide patients and clinicians with information to make informed choices about the risks vs. benefits of treatment with these medications. 

We want to emphasise the possibility of cholangitis as a side effect of ICI treatment as an important differential diagnosis, prompting particularly non-specialist centres to consider early imaging, early discussion with specialist oncology teams and prompt treatment. Lack of awareness of this and delay in immunosuppressive treatment can have potentially serious complications, as this is a condition with a high mortality in an already high-risk group of patients. In one study, 17% of patients with ICI-induced cholangitis died, and the hospital admission rate was 41.2%, suggesting a high morbidity and clinical/cost burden [10].

## Conclusions

This case highlights immune-related cholangitis as a rare type of irAE, and as the use of ICIs is increasing, clinicians should be aware of all immune-related complications, but specifically cholangitis. Discussion of adverse events related to immunotherapy is essential to guide the benefit vs. risks of treatment to aid future recognition and early intervention. The case underscores the need for awareness of this complication, particularly amongst non-specialists at district hospitals, to encourage early discussion with specialist centres for patients who have been on ICI therapy.

The patient in this case provided written consent for publication. When asked about his perspective on his diagnosis, he emphasised the feeling of uncertainty and felt the diagnosis was unexpected. Overall, he felt reassured by the treatment he was receiving and emphasised that he would take comfort in knowing that learning from his condition could help others in the future.

## References

[1] Onoi K, Chihara Y, Uchino J (2020). Immune checkpoint inhibitors for lung cancer treatment: a review. J Clin Med.

[2] Tan H, Ou X, Chen Y, Lan W, Luo L (2025). A pharmacovigilance study of immune checkpoint inhibitor-associated cholangitis: insights from the FDA Adverse Event Reporting System. Hepatol Res.

[3] Pi B, Wang J, Tong Y, Yang Q, Lv F, Yu Y (2021). Immune-related cholangitis induced by immune checkpoint inhibitors: a systematic review of clinical features and management. Eur J Gastroenterol Hepatol.

[4] Fang W, Sun W, Fang W, Zhang J, Wang C (2024). Clinical features, treatment, and outcome of pembrolizumab induced cholangitis. Naunyn Schmiedebergs Arch Pharmacol.

[5] Okamoto K, Hijioka S, Nagashio Y (2024). Immune-related adverse event-associated sclerosing cholangitis due to immune checkpoint inhibitors: imaging findings and treatments. Jpn J Clin Oncol.

[6] Yasuda T, Ito T, Ishikawa T (2025). Clinical features and pathological findings by liver biopsy in patients with immune-related sclerosing cholangitis induced by immune checkpoint inhibitors. Dig Liver Dis.

[7] Onoyama T, Takeda Y, Yamashita T (2020). Programmed cell death-1 inhibitor-related sclerosing cholangitis: a systematic review. World J Gastroenterol.

[8] Liu W, Huo G, Chen P (2023). Clinical benefit of pembrolizumab in treatment of first line non-small cell lung cancer: a systematic review and meta-analysis of clinical characteristics. BMC Cancer.

[9] He BX, Zhong YF, Zhu YB (2022). Deep learning for predicting immunotherapeutic efficacy in advanced non-small cell lung cancer patients: a retrospective study combining progression-free survival risk and overall survival risk. Transl Lung Cancer Res.

[10] Suzuki N, Ikeda Y, Ono M, Ohmori G, Maeda M (2022). Gastrointestinal: Immune-related sclerosing cholangitis with pembrolizumab: imaging and histological features. J Gastroenterol Hepatol.

